# Changes in Cx43 and Na_V_1.5 Expression Precede the Occurrence of Substantial Fibrosis in Calcineurin-Induced Murine Cardiac Hypertrophy

**DOI:** 10.1371/journal.pone.0087226

**Published:** 2014-01-31

**Authors:** Magda S. C. Fontes, Antonia J. A. Raaijmakers, Tessa van Doorn, Bart Kok, Sylvia Nieuwenhuis, Roel van der Nagel, Marc A. Vos, Teun P. de Boer, Harold V. M. van Rijen, Marti F. A. Bierhuizen

**Affiliations:** Division of Heart & Lungs, Department of Medical Physiology, University Medical Center Utrecht, Utrecht, The Netherlands; Leiden University Medical Center, Netherlands

## Abstract

In mice, the calcium-dependent phosphatase calcineurin A (CnA) induces a transcriptional pathway leading to pathological cardiac hypertrophy. Interestingly, induction of CnA has been frequently noticed in human hypertrophic and failing hearts. Independently, the arrhythmia vulnerability of such hearts has been regularly associated with remodeling of parameters determining electrical conduction (expression level of connexin43 (Cx43) and Na_V_1.5, connective tissue architecture), for which the precise molecular basis and sequence of events is still unknown. Recently, we observed reduced Cx43 and Na_V_1.5 expression in 4-week old mouse hearts, overexpressing a constitutively active form of CnA (MHC-CnA model), but the order of events is still unknown. Therefore, three key parameters of conduction (Cx43, Na_V_1.5 and connective tissue expression) were characterized in MHC-CnA ventricles *versus* wild-type (WT) during postnatal development on a weekly basis. At postnatal week 1, CnA overexpression induced cardiac hypertrophy in MHC-CnA. Moreover, protein and RNA levels of both Cx43 and Na_V_1.5 were reduced by at least 50% as compared to WT. Cx43 immunoreactive signal was reduced at week 2 in MHC-CnA. At postnatal week 3, Cx43 was less phosphorylated and RNA level of Cx43 normalized to WT values, although the protein level was still reduced. Additionally, MHC-CnA hearts displayed substantial fibrosis relative to WT, which was accompanied by increased RNA levels for genes previously associated with fibrosis such as *Col1a1*, *Col1a2*, *Col3a1, Tgfb1*, *Ctgf*, *Timp1* and microRNA miR-21. In MHC-CnA, reduction in Cx43 and Na_V_1.5 expression thus coincided with overexpression of CnA and hypertrophy development and preceded significant presence of fibrosis. At postnatal week 4 the alterations in conductional parameters observed in the MHC-CnA model lead to abnormal conduction and arrhythmias, similar to those observed in cardiac remodeling in heart failure patients. The MHC-CnA model, therefore, provides for a unique model to resolve the molecular origin of conductional remodeling in detail.

## Introduction

In human cardiac hypertrophy and heart failure, activation of the calcium-dependent phosphatase calcineurin A (CnA) has been frequently observed [1], [2]. In mice, increased intracellular calcium is known to activate CnA, which binds and dephosphorylates members of the nuclear factor of activated T cells (NFAT) transcription factor family. Subsequently, NFAT translocates from the cytoplasm to the nucleus where it potentiates the transcription of multiple hypertrophic marker genes. Transgenic mice overexpressing a constitutively active form of CnA specifically in cardiomyocytes (MHC-CnA) developed cardiac hypertrophy as early as 18 days postnatally, which to varying extent progressed to failure and sudden death [3].

Electrical impulse conduction in the heart is mainly determined by three key parameters: electrical coupling between cardiomyocytes, excitability of individual cardiomyocytes and connective tissue architecture [4]. These parameters of conduction are mainly mediated by connexin43 (Cx43) (major gap junction protein expressed in the heart), by the sodium channel Na_V_1.5, and by the amount of collagen fibers, respectively. In arrhythmogenic remodeled hearts, abnormalities in any of these parameters of conduction have been frequently observed. Cx43 is usually downregulated, less phosphorylated and/or redistributed from the intercalated disks to the lateral sides of cardiomyocytes [5], [6]. Downregulation of Na_V_1.5 at the protein or RNA level, reduction of peak and increased late sodium current have all been frequently reported, but in contrast also no change in *Scn5a* mRNA, the gene encoding Na_V_1.5, has been observed [7]–[11]. Finally, collagen fiber deposition is usually increased (fibrosis) [12]. The precise molecular basis for these changes and the order of events is still largely unknown.

Previously, we associated CnA activation with reduced expression of Cx43 and Na_V_1.5 and with increased susceptibility for polymorphic ventricular tachyarrhythmias in 4-week old hypertrophic MHC-CnA hearts [13]. This model therefore may serve as an accelerated model for the pathophysiological changes seen in heart failure patients.

In the current study, we have exploited the ventricles of this mouse model further to investigate calcineurin-dependent changes in three key conductional parameters (Cx43, Na_V_1.5 and connective tissue architecture) during postnatal development, in order to visualize the order of adverse events ultimately leading to arrhythmias.

## Materials and Methods

### Ethics Statement

The experimental protocol was performed in accordance with the national guidelines and approved by the local Ethical Animal Experimental Committee (approval number 2011.II.04.065) of the University of Utrecht (Utrecht, The Netherlands). All efforts were made to minimize suffering.

### Animals

Male and female MHC-CnA mice, kindly provided by Dr. E. Olson (University of Texas Southwestern Medical Center, Dallas, TX, USA), were compared with age-matched WT littermates (both C57BL/6 background) at week 0, 1, 2, 3 and 4 after birth. Genotype of mice was determined by polymerase chain reaction (PCR) on DNA isolated from ear or tail biopsy specimens, as described before [13]. Mice were housed under standard conditions with food and water given *ad libitum* and maintained on a 12 h light/dark cycle with controlled temperature and humidity.

### Heart Sampling

Mice were sacrificed directly (0) or 1, 2, 3 and 4 weeks after birth and hearts were rapidly excised and rinsed in cold phosphate buffered saline (PBS). Subsequently the hearts were either immediately frozen in liquid nitrogen (for histology) or the atria were discarded and the ventricles frozen in liquid nitrogen (for real-time quantitative PCR (RT-qPCR) and immunoblotting). Only ventricles (both left and right) were analyzed in all experiments. Tissue samples were stored at –80°C until further use.

### Immunohistochemistry and Histology

For immunohistochemistry and collagen detection, cryosections of 4 different WT and MHC-CnA hearts (four-chamber view, 10 µm thickness) were prepared from different levels of the heart. Immunolabeling was performed as previously described [14], antibodies are listed below. After immunolabeling, sections were analyzed with a widefield microscope (Nikon Eclipse 80 i; Nikon Europe B.V., Amstelveen, The Netherlands) with epifluorescence equipment. To evaluate the amount of collagen content, sections were fixed with 4% paraformaldehyde (in PBS, 30 minutes at room temperature), stained with Picrosirius Red as described previously [15] and visualized with brightfield microscopy (Nikon Eclipse 80 i). An investigator that was blinded to the groups took randomly chosen images from both ventricles of each heart with identical camera settings (gain and exposure time) using NIS Elements BR 3.0 software at 200× magnification. Widefield fluorescence images were deconvoluted using Huygens Essential 4.1 software (Scientific Volume Imaging B.V., Hilversum, The Netherlands) and transformed into 8-bit RGB (i.e. Red Green Blue) stack. Between 36 to 40 images per group (genotype/time point) were used for quantification analysis using ImageJ 1.44o software [16], in which only pixels positive for Cx43/Na_V_1.5/Fibrosis are taken into account by defining a mask with a fixed threshold value in the 255-leveled green channel. The amount of fibrosis was calculated as a percentage of the area of each image (expressed in pixels).

### Immunoblotting

Total cellular protein lysates were isolated from the ventricles and analyzed by immunoblotting as described before [14], [17]. Briefly, 20 µg of protein lysate (40 µg for Na_V_1.5 detection) was separated by 7% or 10% (w/v) sodium dodecyl sulfate-polyacrylamide gel electrophoresis (SDS-PAGE) and subsequently electro-transferred onto a nitrocellulose membrane (Bio-Rad Laboratories, Inc., Hercules, CA, USA). Equal loading of protein was assessed by Ponceau S staining. Proteins were detected after incubation with specific primary and secondary antibodies using a standard ECL procedure (GE Healthcare, Buckinghamshire, United Kingdom). Protein levels were expressed as the ratio of protein of interest/correspondent Ponceau S staining and both were quantified using Image Lab 3.0.1 software (Bio-Rad Laboratories, Inc.).

### Antibodies

As primary antibodies, the following antibodies were used: mouse monoclonal antibodies against Cx43 (1∶250, BD Transduction Laboratories by BD Biosciences, Breda, The Netherlands, immunoblotting), Cx43-NP (1∶500, Invitrogen by Life Technologies Corp., Carlsbad, CA, USA) and N-cadherin (1∶800, Sigma-Aldrich Corp., Saint Louis, MO, USA); rabbit polyclonal antibodies against Cx43 (1∶250, Invitrogen by Life Technologies Corp., immunohistochemistry), Na_V_1.5 (1∶200, Alomone Labs, Jerusalem, Israel, immunoblotting; 1∶100, Sigma-Aldrich Corp., immunohistochemistry) and CnA (1∶500, Merck Millipore, Billerica, Massachusetts, USA); goat polyclonal antibodies against connective tissue growth factor (CTGF) (1∶200, a kind gift of Dr. R. Goldschmeding, Department of Pathology, University Medical Center Utrecht, The Netherlands). Alexa Fluor 594 and fluorescein isothiocyanate (FITC)-conjugated anti-mouse or anti-rabbit whole IgG (1∶250, Jackson ImmunoResearch Europe Ltd., Newmarket, United Kingdom) were used as secondary antibodies for immunohistochemistry; horseradish peroxidase (HRP)-conjugated donkey anti-mouse, anti-rabbit (1∶7000, Bio-Rad Laboratories, Inc.) or anti-goat (1∶7000, Jackson ImmunoResearch Europe Ltd.) as secondary antibodies for immunoblotting.

### Real-Time Quantitative PCR (RT-qPCR)

Total RNA was isolated from WT and MHC-CnA ventricles (n = 4) with TRIzol reagent (Invitrogen by Life Technologies Corp.) and subsequently treated with DNAse I (Promega, Madison, WI, USA) as previously described [18]. One µg of RNA was converted to cDNA with Reverse Transcriptase (Invitrogen by Life Technologies Corp.) according to the manufacturer’s protocol and diluted 10-fold prior to PCR amplification. RT-qPCR was performed for each gene in a MyiQ2 Real-Time PCR Detection System (Bio-Rad Laboratories, Inc.) using Applied Biosystems TaqMan Gene Expression Assays (for specifics see Table S1) (Applied Biosystems by Life Technologies Corp.).

For microRNAs (miRNAs) a similar approach was performed as described above for RNA isolation and treatment. TaqMan miRNA Reverse Transcriptase Kit (Applied Biosystems by Life Technologies Corp.) was used to synthesize cDNA from 10 ng of RNA according to the manufacturer’s protocol and diluted 10-fold prior to PCR amplification. RT-qPCR amplification was performed using TaqMan miRNA-specific primers (for specifics see Table S1) (Applied Biosystems by Life Technologies Corp.).

Experiments were carried out in duplicate. Relative expression of MHC-CnA mRNA and miRNA levels was determined using the 2^−ΔΔC^ *_T_* method and normalized to WT expression levels, as previously described [19]. Glyceraldehyde 3-phosphate dehydrogenase (*Gapdh*) and U6 snRNA (Applied Biosystems by Life Technologies Corp.) (for specifics see Table S1) were used as internal controls for mRNA and miRNA levels, respectively, since their levels did not differ between WT and MHC-CnA samples.

### Statistical Analysis

All results are presented as mean ± standard error of the mean (SEM). Shapiro-Wilk test was used to check for normality of data. Differences among group averages were evaluated using unpaired Student’s t-test (RT-qPCR), confidence interval of the ratio [20] (immunoblotting and immunohistochemistry) or two-way ANOVA followed by Tukey-HSD’s *post-hoc* test for multiple comparisons (hypertrophy and histology). Differences were considered statistically significant if *p*<0.05. Data were analyzed using R version 2.15.1 [21].

## Results

**Figure 1:**
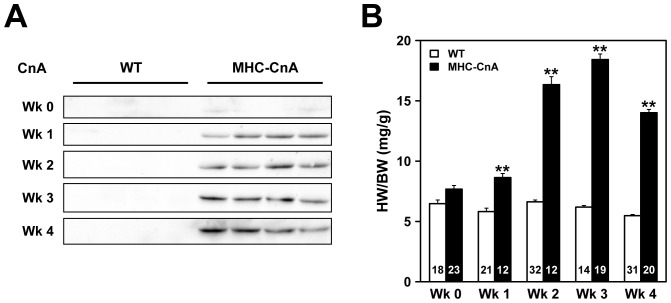
Expression of constitutively active CnA and hypertrophy in WT and MHC-CnA hearts. (A) Protein lysates from four different WT and MHC-CnA ventricles were analyzed for expression of constitutively active CnA (∼43 kDa) by immunoblotting at weeks (Wk) 0, 1, 2, 3 and 4. (B) Hypertrophy is indicated by heart weight/body weight (HW/BW) ratio. Number of mice per group is indicated in the bar graph. Values are mean ± SEM; **p<0.01 compared to WT.

In the present study the development of both structural and conductional remodeling was investigated in MHC-CnA ventricles at weeks 0, 1, 2, 3, and 4 after birth. Transgenic overexpression of CnA, lacking functional and auto-inhibitory domains (∼43 kDa) and driven by the αMHC promotor in this MHC-CnA mouse model, was slightly visible right after birth (week 0) and clearly detectable in the ventricles 1 week after birth and persisted in the following weeks (Figure 1A). In the blot of Figure 1A no bands are visible in WT ventricles, since endogenous CnA is only detectable at a higher molecular mass (∼58 kDa) (Figure S1). Hypertrophy, assessed by heart weight/body weight (HW/BW) ratio, was present at week 1 in MHC-CnA hearts (1.5-fold increase compared to WT; *p*<0.001) and increased in the following weeks as shown in Figure 1B. The slight decrease in HW/BW ratio from week 3 to 4 in MHC-CnA is explained by the smaller increase in HW as compared to BW (Figure S2).

**Figure 2:**
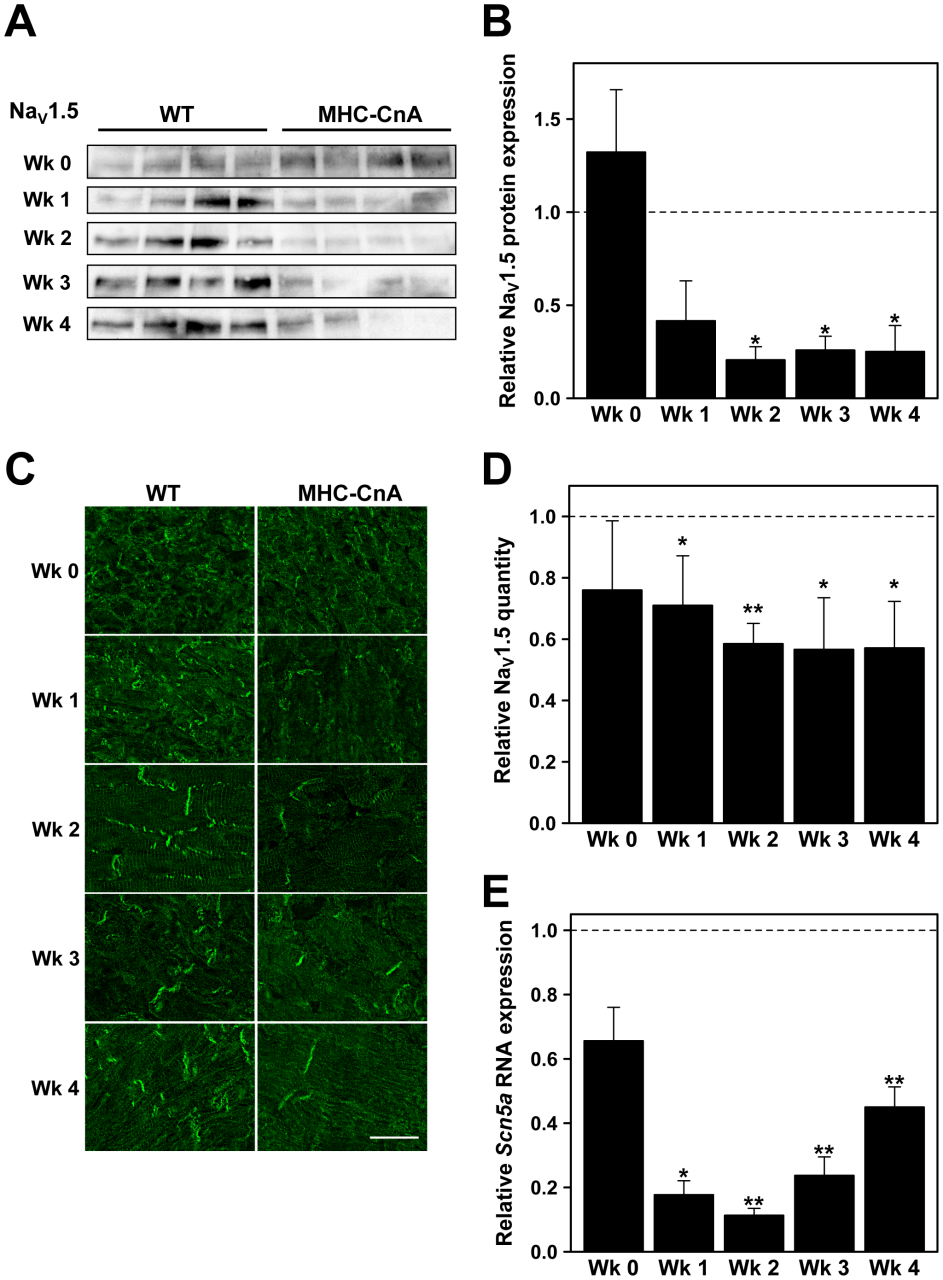
Sodium channel Na_V_1.5 expression in WT and MHC-CnA ventricles. (A) Protein lysates from four different WT and MHC-CnA ventricles were analyzed for Na_V_1.5 expression by immunoblotting at weeks (Wk) 0, 1, 2, 3 and 4. (B) Quantification of the blots (ratio of Protein/Ponceau) represented in (A). (C) Representative images from WT and MHC-CnA ventricles immunolabeled for Na_V_1.5. Scale bar represents 25 µm. (D) Quantification of Na_V_1.5 labeling intensity exemplified in (C). (E) *Scn5a*, the gene encoding Na_V_1.5, was assessed by TaqMan RT-qPCR. MHC-CnA values (B, D and E) are relative to WT (set to 1). Values are mean ± SEM; *p<0.05, **p<0.01 compared to WT.

The protein levels of the sodium channel Na_V_1.5, that primarily mediates excitability in the heart, were assessed with immunoblotting (Figure 2A). At week 0 there was no statistically significant difference between WT and MHC-CnA protein expression in the ventricles. From week 1 until week 4 there was a reduction in Na_V_1.5 protein level in MHC-CnA by more than 50% (58%, 79%, 74% and 75% relative to WT, respectively), although this reduction was only significant from week 2 to 4 (*p*<0.05; Figure 2A and B). The protein levels of Na_V_1.5 were also assessed with immunohistochemistry (Figure 2C). In MHC-CnA, Na_V_1.5 labeling intensity was reduced by 29–43% from week 1 to 4 relative to WT (*p*<0.05; Figure 2C and D), correlating with the reduction detected by immunoblotting (Figure 2A and B). To determine if the reduction in Na_V_1.5 protein expression was accompanied by a reduction in RNA levels, RT-qPCR was performed (Figure 2E). RNA expression for *Scn5a*, the gene encoding Na_V_1.5, revealed a trend towards reduced levels in MHC-CnA already at week 0 (by 34%; *p* = 0.052). However, the reduction in RNA expression was only significant from week 1 until week 4 (reduction by 82%, 89%, 76% and 55% relative to WT, respectively; Figure 2E).

**Figure 3:**
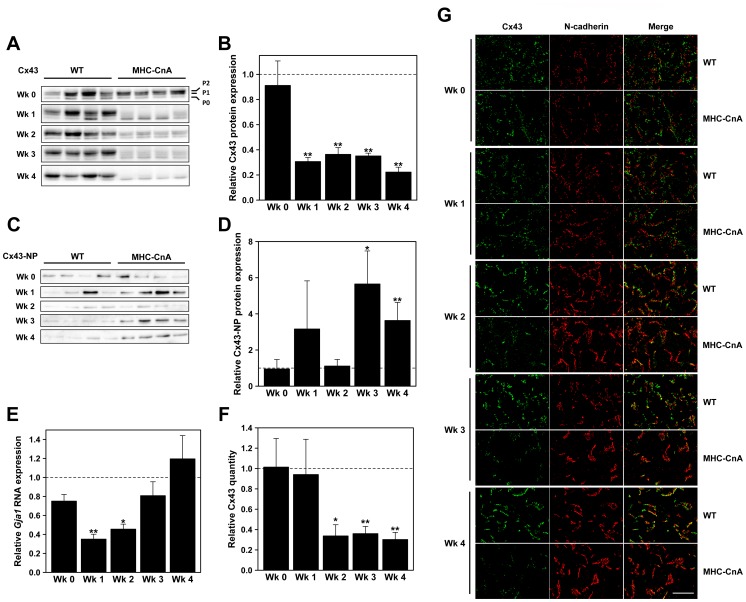
Gap junction Cx43 expression in WT and MHC-CnA ventricles. Protein lysates from four different WT and MHC-CnA ventricles were analyzed for Cx43 (A) and Cx43-NP (C) expression by immunoblotting at weeks (Wk) 0, 1, 2, 3 and 4. In (A) P0, P1 and P2 isoforms are indicated, where P2 corresponds to the most phosphorylated isoform and P0 to the least phosphorylated isoform of Cx43. (B) Quantification of total Cx43 (P0+P1+P2) of the blots (ratio of Protein/Ponceau) represented in (A). In (C) Cx43-NP antibody recognizes the P0 isoform of Cx43 specifically when Ser368 is non-phosphorylated. (D) Quantification of the blots (ratio of Protein/Ponceau) represented in (C). (E) *Gja1*, the gene encoding Cx43, was assessed by TaqMan RT-qPCR. (F) Quantification of Cx43 labeling intensity exemplified in (G). (G) Representative images from WT and MHC-CnA ventricles immunolabeled for Cx43 (green) and N-cadherin (red), a marker for intercalated disk. Scale bar represents 25 µm. MHC-CnA values (B, D, E and F) are relative to WT (set to 1). Values are mean ± SEM; *p<0.05, **p<0.01 compared to WT.

The gap junction protein Cx43, primarily mediating electrical coupling in the heart, was also investigated at the different time points (Figure 3A). The analysis of total Cx43 protein levels revealed no difference between WT and MHC-CnA ventricles right after birth (week 0). From week 1 until week 4 there was a reduction in total Cx43 protein expression (69%, 64%, 65% and 78% relative to WT, respectively; Figure 3A and B). Upon immunoblotting, different Cx43 isoforms can be distinguished on the basis of their phosphorylation status. In Figure 3A, P0 is referred to as the least phosphorylated and P2 as the most phosphorylated isoform of Cx43. Moreover, from these blots it appeared that the Cx43 P0 isoform was relatively increased in some of the weeks relative to the other isoforms in MHC-CnA, suggesting reduced phosphorylation of Cx43. Therefore, we investigated the phosphorylation status of Cx43 using Cx43-NP antibody that recognizes the Cx43 P0 isoform specifically when serine residue 368 (Ser368) is non-phosphorylated (Figure 3C). Using Cx43-NP antibody, an increase in the Cx43 P0 isoform was detected at weeks 3 and 4 in MHC-CnA (by 5.7- and 3.6-fold, respectively). Week 1 also presented higher non-phosphorylated levels (by 3.2-fold), although not significant, possibly due to the unexpected increase in one of the WT samples (Figure 3C and D). Additionally, another antibody that also recognizes the Cx43 P0 isoform (Cx43-CT1 antibody) showed in the same way an increase in P0 isoform at weeks 3 and 4, although week 3 was not significant (Figure S3). To determine if Cx43 RNA levels accompanied the alterations in protein expression, RT-qPCR was performed (Figure 3E). RNA expression for *Gja1*, the gene encoding Cx43, revealed reduced levels in MHC-CnA ventricles (by 25%) at week 0 compared to WT, though not significant (*p* = 0.054). A significant reduction in RNA levels of *Gja1* was detected at weeks 1 and 2 (by 65% and 55%, respectively), increasing back to WT values in the following weeks (Figure 3E). Figure 3G shows typical examples of Cx43 and N-cadherin immunolabeled sections in WT and MHC-CnA ventricles at weeks 0, 1, 2, 3 and 4. From week 0 to 4 the hearts are developing and the cardiomyocytes transiting from a fusiform-like to a more elongated and mature (“brick stone”)-like appearance, as evidenced by the development and formation of the intercalated disks detected with N-cadherin (and Cx43 in the case of the WT hearts). In MHC-CnA, Cx43 labeling intensity was reduced to 30–36% of WT in weeks 2, 3 and 4 (*p*<0.05; Figure 3F and G), but not in weeks 0 and 1.

**Figure 4:**
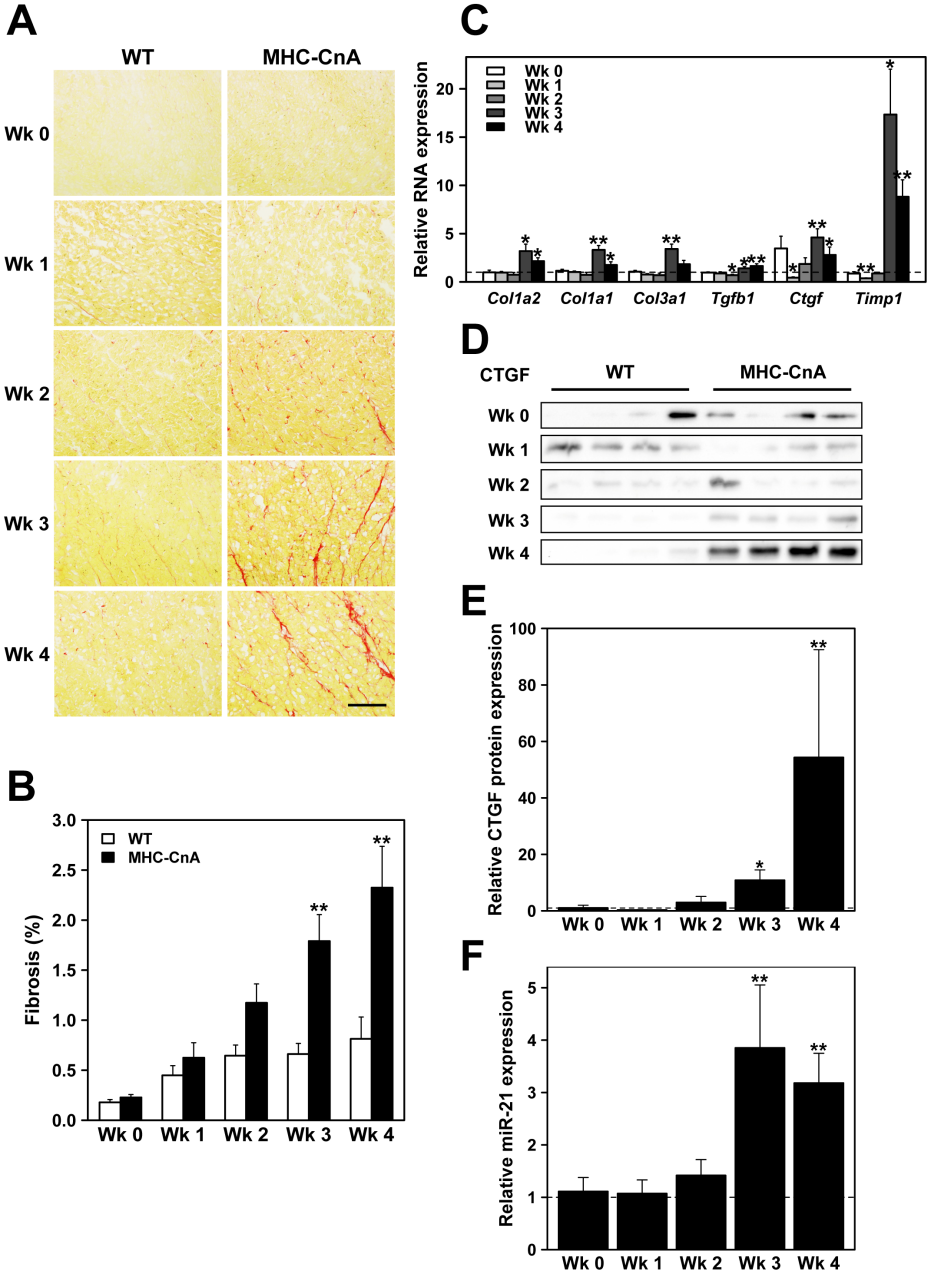
Fibrosis in WT and MHC-CnA ventricles. (A) Picrosirius Red representative images of WT and MHC-CnA ventricles from week (Wk) 0 to 4. Scale bar represents 100 µm. (B) Quantification of Picrosirius Red collagen content. (C) *Col1a1*, *Col1a2*, *Col3a1, Tgfb1*, *Ctgf*, and *Timp1* RNA expression assessed by TaqMan RT-qPCR. (D) Protein lysates from four different WT and MHC-CnA ventricles were analyzed for CTGF expression by immunoblotting. (E) Quantification of the blots (ratio of Protein/Ponceau) represented in (D). MiR-21 miRNA expression assessed by TaqMan RT-qPCR. MHC-CnA values (C, E and F) are relative to WT (set to 1). Values are mean ± SEM; *p<0.05, **p<0.01 compared to WT.

Finally, the amount of ventricular fibrosis, primarily determining tissue architecture in the heart, was also investigated (Figure 4A). Picrosirius Red staining for fibrosis revealed a continuous increase in MHC-CnA ventricles from week 2 until week 4. This increase was only significantly different from WT in weeks 3 and 4 (1.8±0.3% *versus* 0.7±0.1% and 2.3±0.4% *versus* 0.8±0.2%, respectively; *p*<0.01; Figure 4A and B). Interstitial fibrosis found in MHC-CnA ventricles at weeks 3 and 4 was not identified in specific regions of the heart, thus no correlation exists between local fibrosis and reduced Cx43/Na_V_1.5 expression. In line with the enhanced interstitial fibrosis in MHC-CnA hearts, development of mRNA expression levels (from week 0 to 4) of several genes involved in connective tissue formation and degradation were analyzed with TaqMan RT-qPCR assays (Figure 4C). Collagen (*Col1a1*, *Col1a2*, *Col3a1*) RNA levels were similar between WT and MHC-CnA ventricles from week 0 until week 2, whereas in weeks 3 and 4 RNA levels increased. Transforming growth factor beta1 (*Tgfb1*) RNA levels were similar between WT and MHC-CnA at weeks 0 and 1, decreased by 30% in MHC-CnA at week 2 and increased by 1.4- and 1.6-fold, respectively, at weeks 3 and 4. *Ctgf* RNA level was increased in MHC-CnA at week 0 (although not significantly), significantly reduced to 44% of WT at week 1, similar to WT at week 2 and significantly increased by 4.6- and 2.8-fold, respectively, at weeks 3 and 4. Similarly, tissue inhibitor of metalloproteinase 1 (*Timp1*) RNA level in MHC-CnA was not different from WT at week 0, significantly reduced at week 1 to 38% of WT, similar to WT at week 2, and significantly increased by 17.4- and 8.8-fold, respectively, at weeks 3 and 4 (Figure 4C). Interestingly, matrix metallopeptidase 2 (*Mmp2*) and matrix metallopeptidase 9 (*Mmp9*) were not different from WT values at week 4 (data not shown). To determine whether the observed increase of *Ctgf* RNA was correlated with the corresponding alteration at the protein level, immunoblotting was performed (Figure 4D). Quantification of CTGF protein expression revealed similar levels in MHC-CnA compared to WT ventricles at weeks 0 and 2, at week 1 a decrease of 70% (but not significant) and at weeks 3 and 4 a significant 10.8- and 54.3-fold increase, respectively (Figure 4E). Figure 4D shows that WT samples at week 1 had higher CTGF expression than in other weeks probably explaining the reduction observed in MHC-CnA at week 1 (Figure 4C and E). In weeks 3 and 4 CTGF protein and RNA levels correlated well: both were increased. Since four miRNAs were previously implicated in the regulation of fibrosis, namely miR-21, miR29b, miR-30c and miR-133a [12], their expression level was also determined. Since only miR-21 was significantly upregulated in MHC-CnA ventricles at week 4 (data not shown), we further investigated miR-21 in the other weeks. MiR-21 in MHC-CnA was similar to WT from week 0 until week 2 and was significantly increased at weeks 3 and 4 (3.9- and 3.2-fold, respectively; *p*<0.01; Figure 4F), which corresponded to the increase observed in fibrosis (Figure 4A and B).

## Discussion

**Figure 5:**
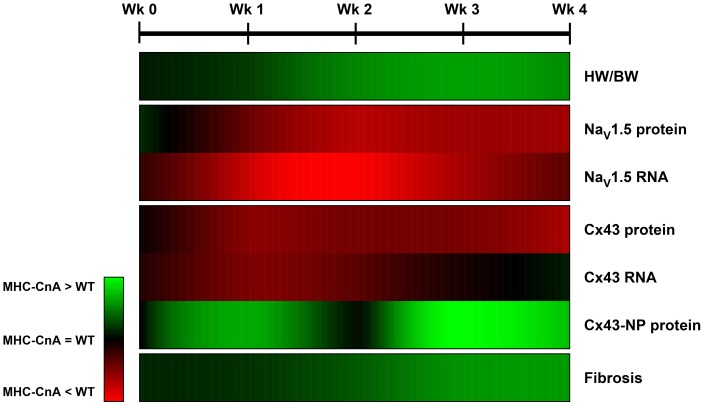
Schematic overview of calcineurin-dependent changes in key conductional parameters of MHC-CnA ventricles during postnatal development. The conductional parameters are listed on the right of the map. Map illustrates the level of upregulation or downregulation based on the color scheme in the legend. MHC-CnA values are relative to WT (set to 1) from week (Wk) 0 to 4 (on the top of the map). Na_V_1.5 and Cx43 protein levels reflect values from immunoblotting; Cx43-NP antibody recognizes the P0 isoform of Cx43 specifically when Ser368 is non-phosphorylated; fibrosis levels reflect values from the Picrosirius Red staining. HW/BW indicates heart weight/body weight ratio.

In this study we targeted the development of conductional remodeling in calcineurin-induced cardiac hypertrophy and newly demonstrated that (in MHC-CnA, Figure 5): 1) overexpression of continuously active CnA and cardiac hypertrophy already appeared at postnatal week 1; 2) the appearance of cardiac hypertrophy coincided with reductions in both Na_V_1.5 and Cx43 protein/RNA expression; 3) the initial reduction in Cx43 RNA (but not protein) expression normalized at postnatal weeks 3 and 4, paralleled by a decrease in Cx43 phosphorylation; 4) fibrosis occurred later than hypertrophy and was significantly detectable from postnatal week 3 onwards, which was further substantiated by increased RNA levels for *Col1a1*, *Col1a2*, *Col3a1, Tgfb1, Ctgf, Timp1* and microRNA miR-21. Overall, many aspects of the conductional remodeling observed in the MHC-CnA model resemble those that have been reported for human cardiac hypertrophy and failure [5]–[12].

The observed association at postnatal weeks 1–4 between Na_V_1.5 protein/RNA downregulation and the expression of continuously active CnA supports our earlier suggestion for a (NFAT-mediated) transcriptional mechanism for its reduction [13]. Although involvement of NFAT in *Scn5a* regulation has not yet been reported and NFAT is considered, often in collaboration with either GATA4 or MEF2, an enhancer of gene transcription it is possible that NFAT regulation of *Scn5a* depends on an as yet unidentified co-factor. One potential candidate for this role could be the T-box (Tbx) transcription factor Tbx5 as it was recently suggested to be involved in the regulation of *Scn5a* expression [22] and its expression was found to be reduced in MHC-CnA hearts from week 1 onwards (results not shown). Interestingly, Guo *et al*. [23] reported a progressive decrease in sodium current density (I_Na_) with age in MHC-CnA hearts, which was however not accompanied by a reduction in Na_V_1.5 protein expression. Although the reason for this discrepancy is not clear, the use of different mouse strains (ICR *versus* C57BL/6), the level of constitutively active CnA expression, the severity of hypertrophy, and the use of different experimental approaches all may have contributed.

As for Na_V_1.5, the observed reduction in Cx43 protein level correlated with downregulation of *Gja1* RNA expression at postnatal weeks 1 and 2 in MHC-CnA. However, at weeks 3 and 4 the RNA level of *Gja1* normalized to WT values, whereas the protein level remained reduced. This suggests that the initial Cx43 reduction is caused at the transcriptional level, whereas at weeks 3 and 4 another mechanism may be responsible. Analysis of the Cx43 phosphorylation status at Ser368 (Cx43-NP) revealed that Cx43 is less phosphorylated at this site in MHC-CnA at weeks 3 and 4, which may mark Cx43 for preliminary degradation. Since Ser368 is a target for protein kinase C (PKC) [24], reduced phosphorylation by PKC may underlie this phenomenon. Alternatively, CnA as a serine/threonine phosphatase itself may be involved in dephosphorylation of Cx43, either directly or indirectly. In line with this, dephosphorylation of Cx43 following myocardial ischemia in rats was inhibited by cyclosporine A, a CnA inhibitor, suggesting involvement of CnA [25]. Moreover, in a test-tube experiment purified CnA indeed proved to be able to dephosphorylate Cx43, immunoprecipitated from mouse heart, at the mentioned Ser residue (Figure S4 and Methods S1). Besides the above-mentioned potential role of (de)phosphorylation, regulation of Cx43 by particular microRNAs may provide for another such mechanism [26]–[28]. Cx43 expression is known to be regulated by two miRNAs, miR-1 and miR-206 [29], however the expression of these miRNAs was not different between MHC-CnA and WT from week 0 to 4 (data not shown). Decreased expression of mechanical junction proteins plakoglobin, plakophilin or N-cadherin leads to downregulation or altered expression of Cx43 and together it could result in enhanced susceptibility to arrhythmias. [30]–[32] However, in 4-week old MHC-CnA hearts, the expression of these proteins was not decreased (data not shown). Taken together, the observed reduction in Cx43 protein level, when *Gja1* RNA level is similar to WT values, is possibly caused by a reduction in the phosphorylation levels which could trigger degradation of Cx43 by an as yet unknown mechanism, or by another pathway yet to be identified.

The increased collagen deposition in MHC-CnA hearts is in agreement with previous results [3], [33]. The increase in cardiac fibrosis can occur by an increase in collagen gene expression or by a decrease in the breakdown of collagen fibers, which is regulated by different proteolytic enzymes, namely MMP2 and MMP9 [34], [35]. Indeed, we showed that RNA expression levels of collagen in MHC-CnA are significantly upregulated at 3 weeks after birth, coinciding with the histological detection of fibrosis in this mouse model. Additionally, the RNA expression levels of TGF-β1, a known inducer of fibrosis, and CTGF, a known downstream protein of TGF-β [36], [37], were increased in MHC-CnA ventricles at weeks 3 and 4 also coinciding with the histological detection of fibrosis. However, in MHC-CnA the RNA levels of *Mmp2* and *Mmp9* did not differ statistically from WT values. Instead, an upregulation of *Timp1*, a known inhibitor of MMPs [34], coincided with occurrence of fibrosis.

Expression analysis of miR-21, miR-29b, miR-30c and miR-133a (all previously implicated in the regulation of fibrosis [38]–[41]) revealed that the upregulation of miR-21 at weeks 3 and 4 was the only alteration observed between MHC-CnA and WT. This upregulation of miR-21 was correlated with the histological detection of fibrosis. Recently, an increase in miR-21 was also found in patients with atrial fibrillation or heart failure and in mice with pressure overload or cardiac stress [38], [42]. However, the role of miR-21 in fibrosis development is controversial since it has been disputed by two different groups [38], [43]. Interestingly, MHC-CnA mice interbred with miR-21 deficient mice developed cardiac hypertrophy at the same level as MHC-CnA mice, but unfortunately the level of fibrosis was not compared [43]. Altogether, the changes in collagen, TIMP-1, TGF-β1, CTGF and miR-21 expression observed in the MHC-CnA model correlate and may contribute to the increased collagen deposition detected in these hearts.

Recently it was proposed that reduction in Cx43 expression precedes the development of fibrosis in aging or pressure-overloaded mice hearts [44]. Interestingly, in the MHC-CnA mouse model increased collagen deposition occurred after the reduction in Cx43 expression. The ability of individual cardiomyocytes to establish gap junctional communication with fibroblasts has been shown in cell cultures, but also in multicellular tissue over extended distances [45]. This coupling between cardiomyocytes and fibroblasts by Cx43 could potentially be involved in the triggering of collagen deposition; however, the molecular mechanism by which Cx43 reduction may result in excessive fibrosis remains to be elucidated. This combination of reduced Cx43 with excessive fibrosis led to increased susceptibility for ventricular arrhythmias [44]. Another arrhythmia combination is Na_V_1.5 reduction in senescence mice together with the occurrence of extensive fibrosis in a later stage [46].

One of the limitations with transgenic models using the α-MHC promoter is that it can result in the expression of relatively high levels of transgenic protein, the normal variant of which is expressed at lower levels. Another limitation is that the transgene may already be expressed before birth and thus influence cardiac development. In addition, when cardiac hypertrophy/failure has developed the expression of the transgene may decrease due to the α-MHC to β-MHC shift. The limitation of quantifying immunohistological images is the fact that this is a subjective method. The quantification of Cx43 labeling intensity (or collagen content) is performed by the use of a threshold that is set equal to all images and the drawback is there might be some images that can be over- or underestimated by the use of this threshold.

In summary, the reduction of Cx43 and Na_V_1.5 expression coincided with activation of the CnA/NFAT pathway and hypertrophy development, and preceded significant presence of fibrosis. At 4 weeks of age the alterations in conductional parameters observed in the MHC-CnA model lead to abnormal conduction and arrhythmias, similar to those observed in pathophysiological cardiac remodeling in heart failure patients.

## Supporting Information

Figure S1 **Expression of constitutively active CnA in WT and MHC-CnA hearts at week 2.** Protein lysates from four different WT and MHC-CnA ventricles were analyzed for expression of endogenous (∼58 kDa) and exogenous (∼43 kDa; constitutively active) CnA by immunoblotting.
(TIF)

Figure S2 **Heart weight (HW) and body weight (BW) in WT and MHC-CnA mice in weeks (Wk) 0, 1, 2, 3 and 4.**
(TIF)

Figure S3 **Gap junction Cx43 expression in WT and MHC-CnA ventricles.** Protein lysates from four different WT and MHC-CnA ventricles were analyzed for Cx43-CT1 expression by immunoblotting at weeks (Wk) 0, 1, 2, 3 and 4. In (A) Cx43-CT1 antibody recognizes the P0 isoform of Cx43 specifically when Ser364 and/or Ser365 are non-phosphorylated. (B) Quantification of the blots (ratio of Protein/Ponceau) represented in (A). MHC-CnA values are relative to WT (set to 1). Values are mean ± SEM; **p<0.01 compared to WT.
(TIF)

Figure S4 **Cx43 immunoprecipitated from adult mouse heart treated with CnA or with CnA and a CnA inhibitor.** (A) CnA (PP2B) treatment of Cx43 was analyzed at different concentrations for total Cx43 and Cx43 P0 isoform expression by immunoblotting. Cx43-NP antibody recognizes the P0 isoform of Cx43 specifically when Ser368 is non-phosphorylated. (B) Treatment of Cx43 with PP2B or PP2B together with a CnA inhibitor (FK506/FKBP) was analyzed for total Cx43 and Cx43 P0 isoform expression by immunoblotting. Cx43-CT1 antibody recognizes the P0 isoform of Cx43 specifically when Ser364 and/or Ser365 are non-phosphorylated.
(TIF)

Table S1 **References of the Applied Biosystems assays used in this study.**
(PDF)

Methods S1 **Additional methods of co-immunoprecipitation assay and PP2B dephosphorylation assay with supporting references.**
(PDF)
