# Supplementary figures and images for: Changes in Cx43 and NaV1.5 Expression Precede the Occurrence of Substantial Fibrosis in Calcineurin-Induced Murine Cardiac Hypertrophy
Source: PLoS One. 2014 Jan 31;9(1):e87226. doi: 10.1371/journal.pone.0087226 (PMC3909068; doi:10.1371/journal.pone.0087226)

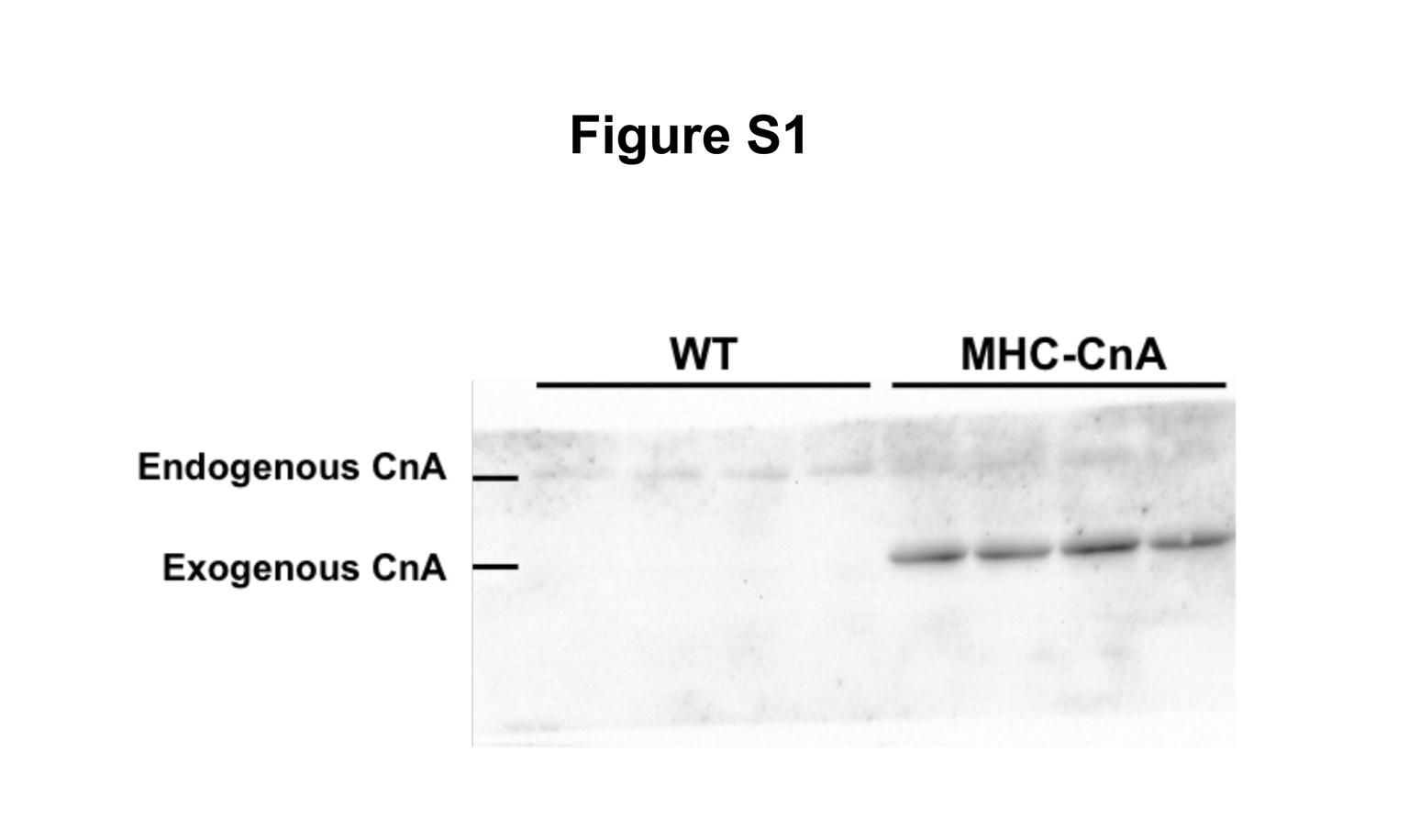

Supplement: Figure S1 — Expression of constitutively active CnA in WT and MHC-CnA hearts at week 2. Protein lysates from four different WT and MHC-CnA ventricles were analyzed for expression of endogenous (∼58 kDa) and exogenous (∼43 kDa; constitutively active) CnA by immunoblotting. (TIF) [file pone.0087226.s001.tif]

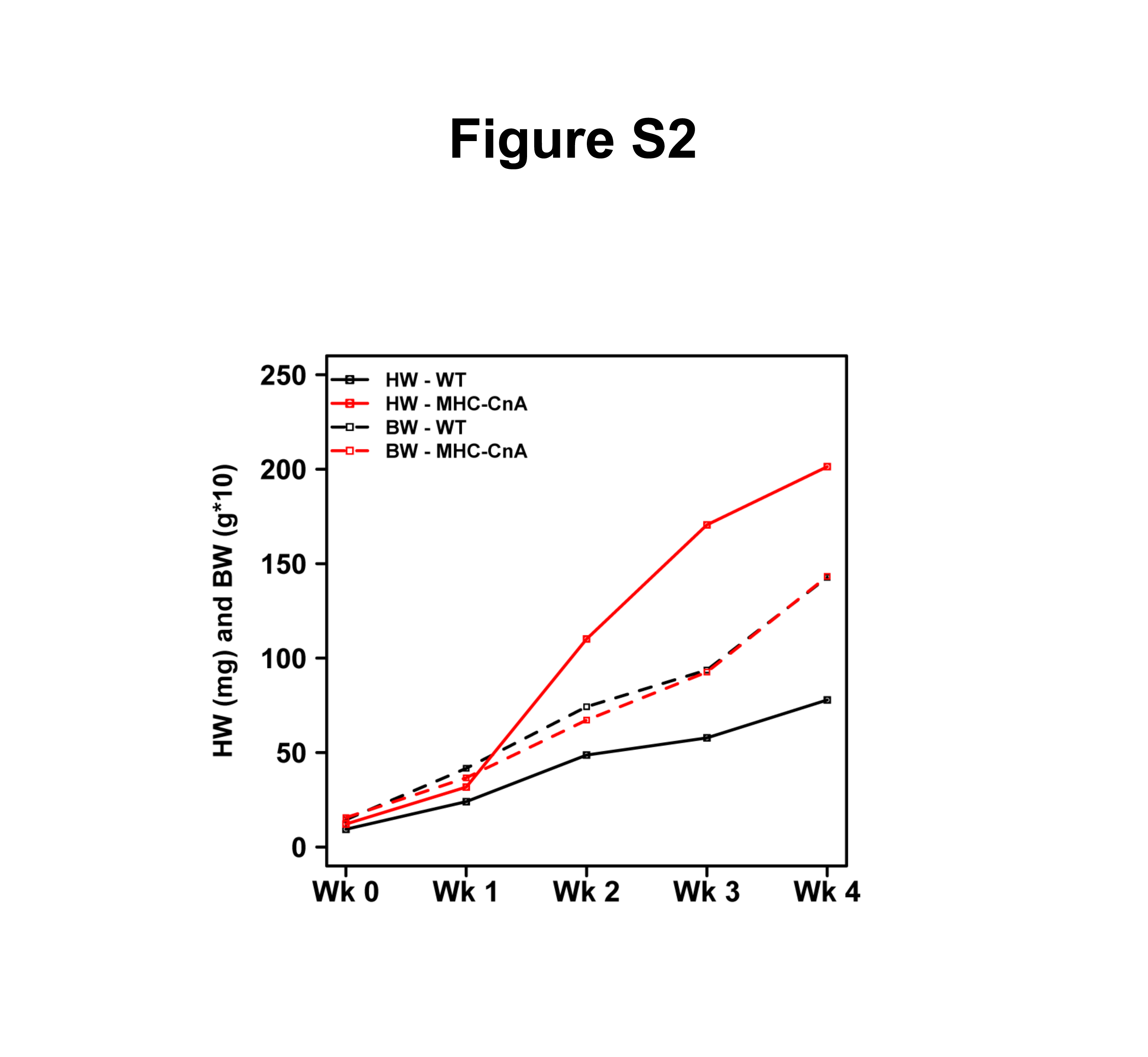

Supplement: Figure S2 — Heart weight (HW) and body weight (BW) in WT and MHC-CnA mice in weeks (Wk) 0, 1, 2, 3 and 4. (TIF) [file pone.0087226.s002.tif]

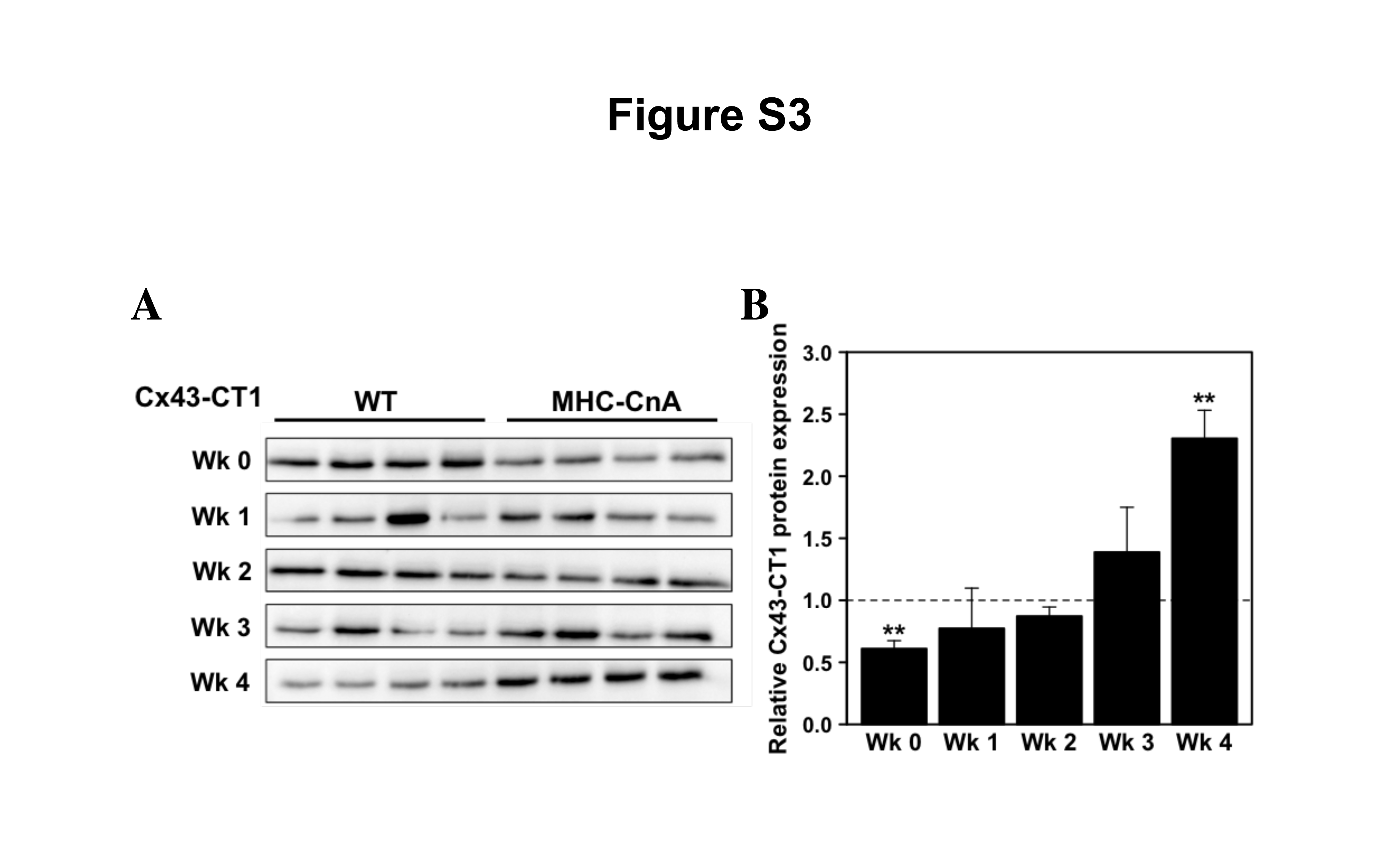

Supplement: Figure S3 — Gap junction Cx43 expression in WT and MHC-CnA ventricles. Protein lysates from four different WT and MHC-CnA ventricles were analyzed for Cx43-CT1 expression by immunoblotting at weeks (Wk) 0, 1, 2, 3 and 4. In (A) Cx43-CT1 antibody recognizes the P0 isoform of Cx43 specifically when Ser364 and/or Ser365 are non-phosphorylated. (B) Quantification of the blots (ratio of Protein/Ponceau) represented in (A). MHC-CnA values are relative to WT (set to 1). Values are mean ± SEM; **p<0.01 compared to WT. (TIF) [file pone.0087226.s003.tif]

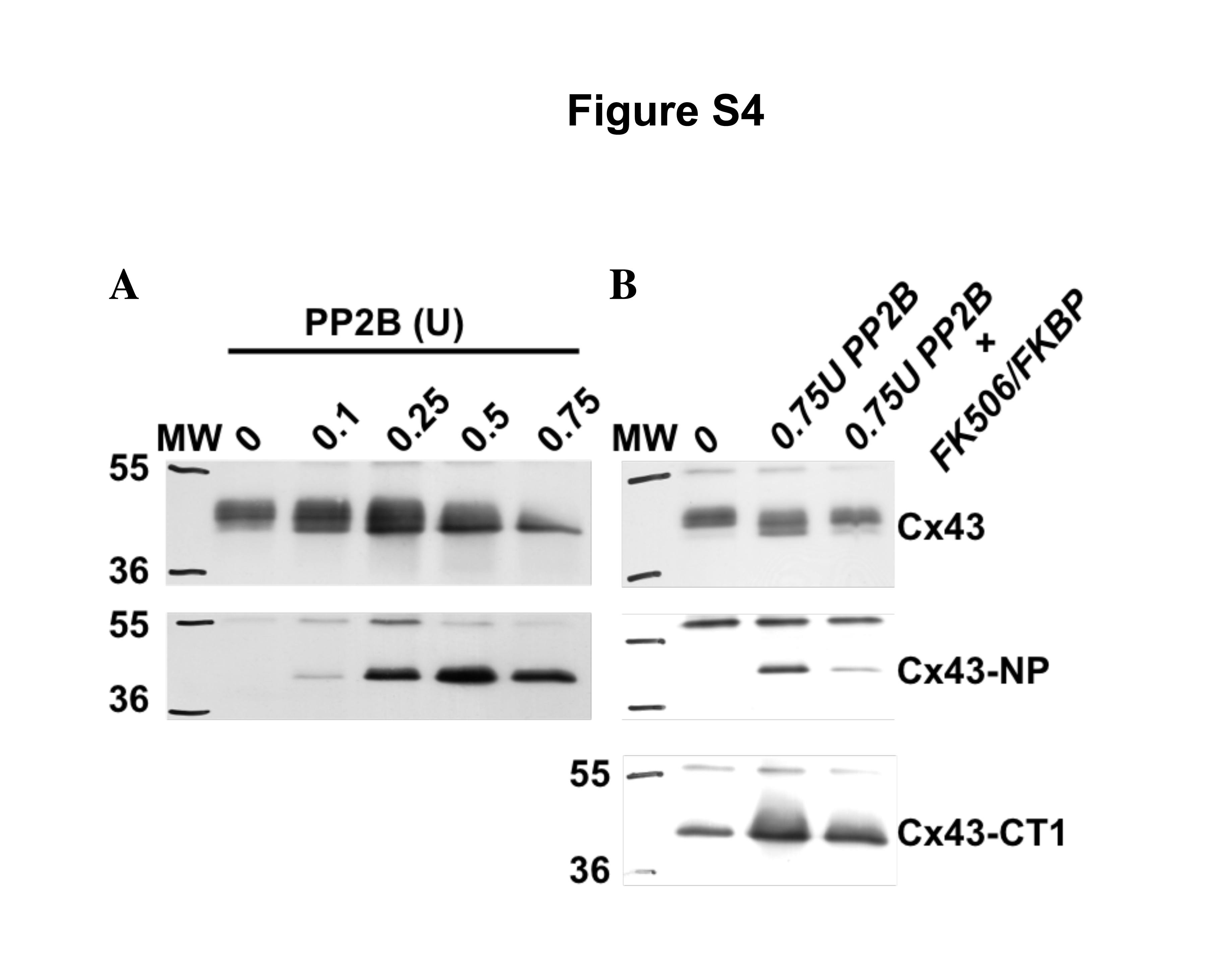

Supplement: Figure S4 — Cx43 immunoprecipitated from adult mouse heart treated with CnA or with CnA and a CnA inhibitor. (A) CnA (PP2B) treatment of Cx43 was analyzed at different concentrations for total Cx43 and Cx43 P0 isoform expression by immunoblotting. Cx43-NP antibody recognizes the P0 isoform of Cx43 specifically when Ser368 is non-phosphorylated. (B) Treatment of Cx43 with PP2B or PP2B together with a CnA inhibitor (FK506/FKBP) was analyzed for total Cx43 and Cx43 P0 isoform expression by immunoblotting. Cx43-CT1 antibody recognizes the P0 isoform of Cx43 specifically when Ser364 and/or Ser365 are non-phosphorylated. (TIF) [file pone.0087226.s004.tif]
