# Supplementary material for: Changes in Cx43 and NaV1.5 Expression Precede the Occurrence of Substantial Fibrosis in Calcineurin-Induced Murine Cardiac Hypertrophy
Source: PLoS One. 2014 Jan 31;9(1):e87226. doi: 10.1371/journal.pone.0087226 (PMC3909068; doi:10.1371/journal.pone.0087226)
Supplement: Table S1 — References of the Applied Biosystems assays used in this study. (PDF) [file pone.0087226.s005.pdf]

Table S1 – References of the Applied Biosystems assays used in this study.

**Gene**	**Assay ID**
Gapdh	Mm99999915_g1
Scn5a	Mm00451971_m1
Gja1	Mm00439105_m1
Col1a1	Mm00801666_g1
Col1a2	Mm00483888_m1
Col3a1	Mm01254476_m1
Ctgf	Mm01192933_g1
Tgfb1	Mm01178820_m1
Timp1	Mm00441818_m1
**miRNA**	**Assay ID**
U6 snRNA	001973
hsa-miR-21	000397
