# Supplementary material for: Changes in Cx43 and NaV1.5 Expression Precede the Occurrence of Substantial Fibrosis in Calcineurin-Induced Murine Cardiac Hypertrophy
Source: PLoS One. 2014 Jan 31;9(1):e87226. doi: 10.1371/journal.pone.0087226 (PMC3909068; doi:10.1371/journal.pone.0087226)
Supplement: Methods S1 — Additional methods of co-immunoprecipitation assay and PP2B dephosphorylation assay with supporting references. (PDF) [file pone.0087226.s006.pdf]

Fontes et al: *Changes in Cx43 and $Na_V1.5$ Expression Precede the Occurrence of Substantial Fibrosis in Calcineurin-Induced Murine Cardiac Hypertrophy.*

Methods S1

Co-immunoprecipitation assay

The co-immunoprecipitation of Cx43 from an adult mouse heart was performed as described previously [1]. Briefly, heart lysates from a pool of 3 adult mouse hearts were homogenized in lysis buffer and centrifuged at 14000 rpm for 10 min. Immunoprecipitation was performed using 12.5 mg of protein lysate as input. To pre-clear the lysate, the supernatant was exposed to protein A/G agarose beads (Roche Applied Science, Indianapolis, IN, USA) for 1 hour at 4°C, followed by centrifugation at 14000 rpm for 1 min at 4°C. The pre-cleared supernatant was incubated for 4 hours at 4°C with 7 µg of mouse anti-Cx43 (BD Transduction Laboratories by BD Biosciences, Breda, The Netherlands) and exposed to 500 µl protein A/G agarose beads overnight at 4°C. After centrifugation, the antibody-protein A/G agarose complex was washed 5 times in lysis buffer to clear the solvent of unbound material. The final pellet was resuspended in 500 µl of lysis buffer and 20 µl was used for each PP2B dephosphorylation assay.

PP2B dephosphorylation assay

For CnA (PP2B) dephosphorylation assays, two individual experiments were performed. One set of tubes was pre-incubated for 10 min at 30°C with PP2B buffer (Merck Millipore, Billerica, Massachusetts, USA), calmodulin (3.3 nM, Merck Millipore) and a range of PP2B enzyme concentrations (0, 0.1, 0.25, 0.5 and 0.75 Units, Merck Millipore). The other set of tubes was pre-incubated for 10 min at 30°C

with PP2B buffer (Merck Millipore), calmodulin (3.3 nM, Merck Millipore) and PP2B enzyme (0.75 Units, Merck Millipore) with or without FK506 (100 µM, Sigma-Aldrich Corp., Saint Louis, MO, USA) and FKBP (10 µM, Sigma-Aldrich Corp.). Pre-incubated solution was added to 20 µl of the immunoprecipitated beads per reaction, incubated for 20 min at 30°C with 800 rpm shaker and centrifuged for 1 min at 1000 rpm. The final pellet was resuspended in 50 µl of blue sample buffer and the protein of interest detected by immunoblotting (see Materials and Methods). The antibodies used for protein detection were mouse monoclonal antibodies against total Cx43 (1:250, BD Transduction Laboratories by BD Biosciences), Cx43-NP (1:500, Invitrogen by Life Technologies Corp., Carlsbad, CA, USA) and Cx43-CT1 (1:500, kindly provided by Dr. P.D. Lampe, Molecular Diagnostics Program, Fred Hutchinson Cancer Research Center, USA) [2].

References

1. Sato PY, Musa H, Coombs W, Guerrero-Serna G, Patiño GA, Taffet SM, Isom LL, Delmar M (2009) Loss of plakophilin-2 expression leads to decreased sodium current and slower conduction velocity in cultured cardiac myocytes. Circ Res 105: 523-526.

2. Sosinsky GE, Solan JL, Gaietta GM, Ngan L, Lee GJ, Mackey MR, Lampe PD (2007) The C-terminus of connexin43 adopts different conformations in the Golgi and gap junction as detected with structure-specific antibodies. Biochem J 408: 375-385.
